# Idiopathic hypogonadotropic hypogonadism caused by compound heterozygosity for two novel mutations in the *GNRH1* gene: a case report

**DOI:** 10.1186/s12902-023-01455-7

**Published:** 2023-10-05

**Authors:** Qingqing Tian, Jingjing Tang, Lihong Wang, Jiaojiao Liu, Xiangshan Li, Zhuozhuo Cao, Zhufang Tian

**Affiliations:** 1 Department of Endocrinology, Xi’an Central Hospital, No. 161 Xiwu Road, Xi’an, 710003 Shaanxi China; 2 Medical School of Yan’an University, Yan’an, 716000 Shaanxi China

**Keywords:** Idiopathic hypogonadotropic hypogonadism, Gonadotropin-releasing hormone 1, Delayed puberty

## Abstract

**Background:**

Idiopathic hypogonadotropic hypogonadism (IHH) is a rare congenital or acquired genetic disorder caused by gonadotropin-releasing hormone (GnRH) deficiency. IHH patients are divided into two major groups, hyposmic or anosmic IHH (Kallmann syndrome) and normosmic IHH (nIHH), according to whether their sense of smell is intact. Here we report a case of novel compound heterozygous mutations in the *GNRH1* gene in a 15-year-old male with nIHH.

**Case presentation:**

The patient presented typical clinical symptoms of delayed testicular development, with testosterone < 3.5 mmol/L and reduced gonadotropin (follicle-stimulating hormone, luteinizing hormone) levels. Two heterozygous variants of the GNRH1 gene were detected, nonsense variant 1: *c.85G* > T:p.G29* and variant 2: *c.1A* > *G:p.M1V*, which disrupted the start codon.

**Conclusions:**

Two *GNRH1* mutations responsible for nIHH are identified in this study. Our findings extend the mutational spectrum of *GNRH1* by revealing novel causative mutations of nIHH.

## Background

Congenital impairment of hypothalamic neurons that produce gonadotropin-releasing hormone (GnRH) affects the synthesis, secretion, or functions of GnRH. This results in reduced secretion of gonadotropin by the pituitary gland and, consequently, idiopathic hypogonadotropic hypogonadism (IHH), which is a type of gonadal insufficiency [1]. The prevalence of IHH is 1–10 per 100,000 individuals [2], and it has a male-to-female ratio of 3–5:1 [3]. IHH patients are divided into two major groups, hyposmic or anosmic IHH (Kallmann syndrome) and normosmic IHH (nIHH), according to whether their sense of smell is intact or not. To date, more than 30 genes have been found to be associated with IHH, including *ANOS1*, *GNRH1*/*GNRHR*, *FGF8*/*FGFR1*, *PROK2*/*PROKR2*, *SEMA3A*/*PLXNA1*, and *KISS1*/*KISS1R* [4]. In particular, *GNRH1* is a clear candidate gene for IHH. However, it was not until 2009 that Chan et al. [5] for the first time reported IHH due to homozygous frameshift mutations in the *GnRH1* gene. They identified homozygous frameshift mutations and four rare heterozygous sequence variants in the *GNRH1* gene among 310 patients with nIHH. To further explore the *GNRH1* mutations, Chan et al. summarized and analyzed the gene sequences of 600 GnRH-deficient patients. Among the patients with nIHH, Kallmann syndrome, and constitutional delay of growth and puberty (CDGP), only two homozygous *GNRH1* mutations were found. The incidence of GnRH receptor gene mutations in normosmic GnRH deficiency is 0.33% [6]. The prevalence of *GNRH1* mutations in the general population is expected to be even lower. In this paper, we report a case of two novel compound heterozygous mutations in the *GNRH1* gene in a patient with nIHH. Based on clinical findings and literature review, recommendations are provided for the clinical management of IHH.

## Case presentation

A boy was found by his parents to have a small penis at the age of 5 years. He was diagnosed with cryptorchidism and micropenis upon admission to a local hospital. He underwent no endocrinological tests at diagnosis and received surgical treatment for right cryptorchidism. When the patient was 13 years old, his penis was still small. He consulted a hospital, but underwent no treatment. At 15 years of age, his penis and testicles were almost the same size as in early childhood. Due to the delayed development of secondary sexual characteristics, the patient had low self-esteem and therefore visited the outpatient clinic of our hospital to seek for clinical treatment. He looked younger than his chronological age, with no beard or pubic hair, no change of voice, no seminal emission, and no loss of smell or vision. His sex hormone levels were examined at the outpatient clinic: prolactin, 3.58 ng/mL (normal range (NR): 2.64–13.13 ng/mL); luteinizing hormone (LH), 0.38 IU/L (NR: 1.24–8.62 IU/L); follicle-stimulating hormone (FSH), 0.32 IU/L (NR: 1.27–19.26 IU/L); estradiol, 7.13 pg/mL (NR: 15–38.95 pg/mL); and testosterone, 0.42 ng/mL (NR: 1.75–7.81 ng/mL). Based on these findings, IHH was tentatively considered and hospitalization was recommended for ongoing consultation and treatment. His birth weight was 3500 g, and his mother had a normal full-term delivery. He was born of non-consanguineous parents who were both alive, and he had a healthy sister.

**Fig. 1:**
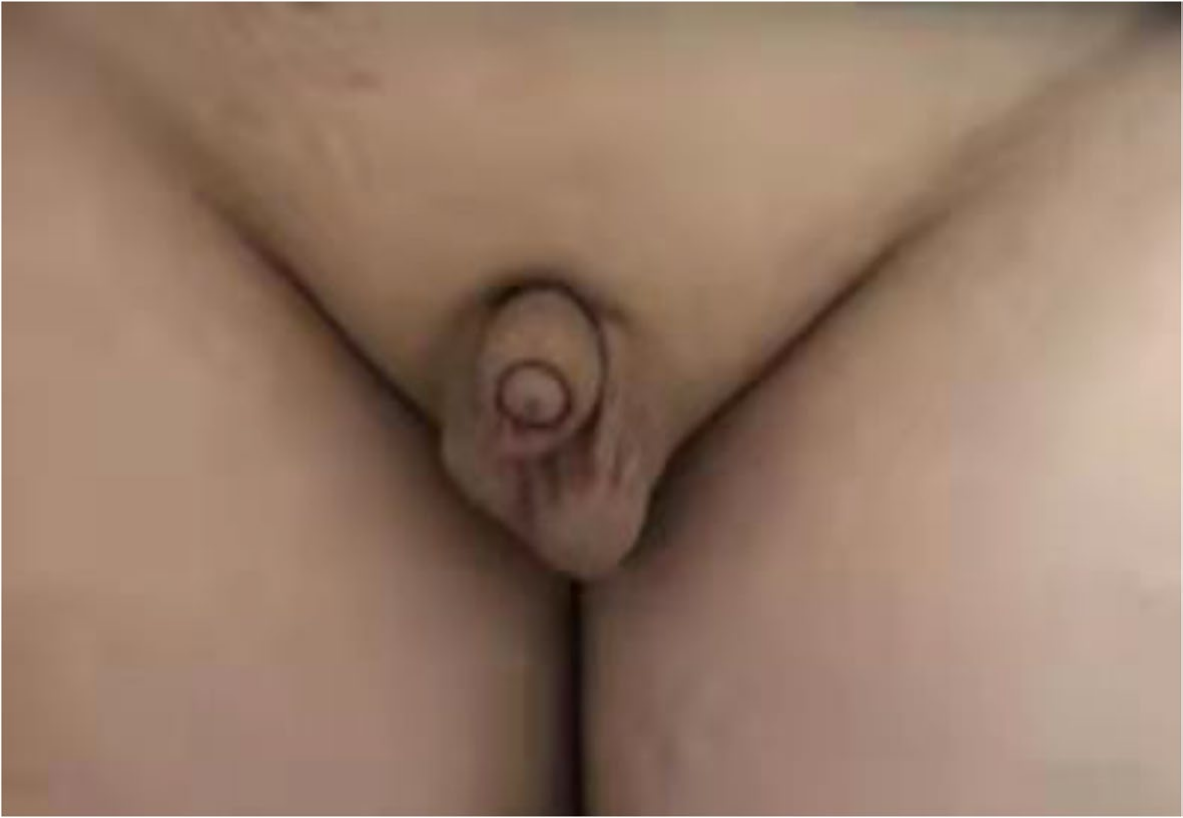
A photo showing the genital appearance of the 15-year-old patient with idiopathic hypogonadotropic hypogonadism at hospital admission. The size and proportions of the testicles, scrotum and penis are approximately the same as in early childhood, and there is no pubic hair. This is classified as Tanner stage I

A physical examination revealed that the patient had a body height of 169 cm and his body weight was 69 kg, with a body mass index of 24.2 kg/m^2^. His upper body segment was 63 cm and the lower body segment was 87 cm, with an arm span of 163 cm. His thyroid gland was enlarged (grade II), with a soft texture and no pressure pain. No noticeable abnormalities were found in the heart, lungs, or abdomen. His intelligence was normal, with no gingival defects and no cleft lip or palate. He had normal visual field and sense of smell (e.g., alcohol, vinegar), with no gynecomastia and no beard, axillary, or pubic hair. His penis was 3 cm long and 7 cm in circumference, which was classified as Tanner stage I (Fig. 1). Tanner stage 1 is characterized by the absence of pubic hair, with the scrotum and penis being about the same size and proportion as in early childhood [7].

Routine blood test of the patient showed low hemoglobin level at 119 g/L (NR: 130–175 g/L), and routine urine test indicated weak positivity for urinary protein. Liver function test revealed increased levels of aspartate aminotransferase (49 U/L, NR: 15–40 U/L) and alkaline phosphatase (253 U/L, NR: 45–125 U/L). Blood lipid test showed high level of triglycerides at 2.11 mmol/L (NR: 0–1.7 mmol/L). The levels of renal enzymes, electrolytes, myocardial enzymes, and coagulation factors were normal. Electrocardiogram showed left ventricular high voltage. A plain computed tomography (CT) scan of the chest indicated an old fracture of the right seventh rib and fatty liver with intrahepatic foci of calcification. An abdominal CT scan revealed fatty liver and multiple small lymph nodes in the right lower abdominal cavity. An X-ray bone age test showed that the bone age of the left wrist was equivalent to 13 years old, while his actual age was 15 years old. Abdominal ultrasound examination detected fatty liver, but no significant abnormalities were found in the biliopancreatic area, both kidneys, or either prostate gland. Thyroid ultrasound examination revealed bilateral thyroid lobe cysts (TI-RADS2 level; left lobe: 0.5 × 0.3 cm, right lobe: 0.3 × 0.2 cm), with bilateral enlarged lymph nodes in zone II of the neck. Testicular ultrasound examination revealed paratesticular effusion on the right side and small testicular volume on both sides (left testicle: 1.1 × 0.7 × 1.5 cm, right testicle: 1.1 × 0.6 × 1.5 cm). Bone mineral density test showed Z values of –1.9 (lumbar spine) and –1.0 (left hip). The thyroid function test results were as follows: thyroid-stimulating hormone, 2.44 µIU/mL (NR: 0.27–4.2 µIU/mL); tri-iodothyronine, 2.56 nmol/L (NR: 1.2–3.1 nmol/L); thyroxine, 132 nmol/L (NR: 59–154 nmol/L); insulin-like growth factor 1, 217.6 ng/mL (NR at 15 years of age, 235–988 ng/mL); growth hormone, 0.078 ng/mL (NR of male: 11–17 years, 0.077–1.08 ng/mL); adrenocorticotropic hormone (08:00 am), 12.47 pg/mL (NR: 7.2–63.4 pg/mL); cortisol (08:00 am), 8.10 µg/mL (NR: 4.26–24.85 µg/mL); cortisol (16:00), 4.78 µg/mL (NR: 2.9–17.3 µg/mL); and cortisol (24:00), 0.69 µg/mL (NR: 0–0.672 µg/mL). The height of the pituitary measured by magnet resonance imaging (MRI) was 2 mm. Re-examination revealed decreased levels of growth hormone (0.039 ng/mL, NR: 11–17 years, 0.077–1.08 ng/mL) and insulin-like growth factor 1 (218.87 ng/mL, NR at 15 years of age: 235–988 ng/mL). Gonadotropin stimulation test showed that the basal LH was 0.55 IU/L, with a peak value of 2.38 IU/L at 60 min after intravenous injection of 100 µg gonadorelin (Table 1). In the human chorionic gonadotropin (HCG) test, the basal testosterone level was 0.03 ng/mL, and after injection of HCG at a dose of 2000 IU, it increased to 0.11 ng/mL at 24 h and 0.22 ng/mL at 72 h, followed by a sharp decrease to 0.13 ng/mL at 96 h (Table 2). Karyotype analysis revealed 46XY karyotype.

**Table 1:** Result of gonadotropin-releasing hormone stimulation test from the patient with normosmic idiopathic hypogonadotropic hypogonadism

Hormone	0 min	15 min	30 min	45 min	60 min	90 min	120 min
Luteinizing hormone (IU/L)	0.55	1,71	2.33	2.57	2.38	1.85	1.79
Follicle-stimulating hormone (IU/L)	0.44	1.64	2.33	3.25	3.64	3.76	3.26

**Table 2:** Result of gonadotropin stimulation test from the patient with normosmic idiopathic hypogonadotropic hypogonadism

Hormone	0 h	24 h	48 h	72 h	96 h
Dehydroepiandrosterone (ng/mL)	1.98	1.63	1.88	2.49	2.50
Testosterone (ng/mL)	0.03	0.11	0.19	0.22	0.13

The main clinical manifestations of the patient were lack of development of secondary sexual characteristics, low levels of sex hormones, and lagging of bone age. Accordingly, the diagnosis of hypergonadotropic hypogonadism was first ruled out. Hypogonadism may arise from testicular disease or dysfunction of the hypothalamic-pituitary unit [8] Subsequent tests of insulin-like growth factor 1, thyroid-stimulating hormone, adrenocorticotropic hormone, growth hormone, prolactin, pituitary MRI, bone density, and bone age were all normal. This indicates that the patient had normal function of anterior pituitary hormone secretion, except for the impaired function of hypothalamic-pituitary–gonadal axis. Then, gonadotropin stimulation test was conducted to exclude the diagnosis of CDGP. Further, HCG test and testicular ultrasonography were carried out to exclude primary testicular disease. All pieces of evidences were indicative of IHH, and the patient had normal olfactory function. Therefore, the final diagnosis was IHH with normal olfactory function (nIHH). As such, the patient was started on testosterone replacement therapy. Testosterone undecanoate was given orally at a dose of 40 mg once a day in the beginning, and it was adjusted to 40 mg twice a day 1 week later. One month later, the dose was adjusted to 40 mg three times a day, and the patient has been continuing with this dose.

**Fig. 2:**
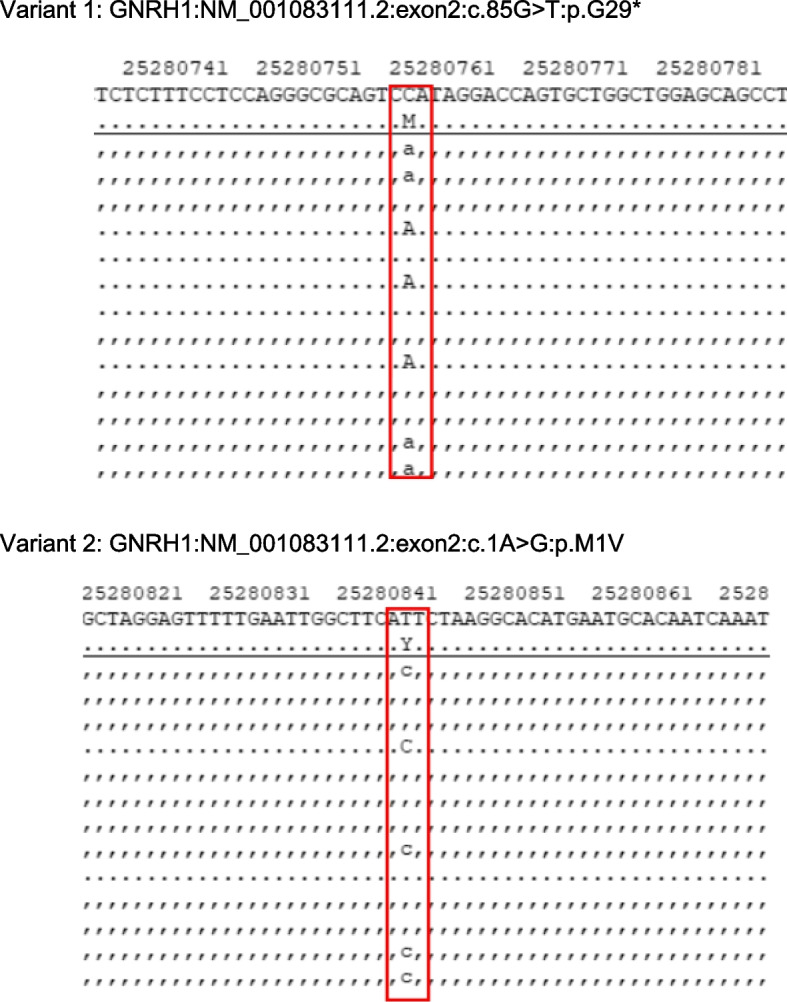
Two mutations identified by whole-exome sequencing in the patient

**Fig. 3:**
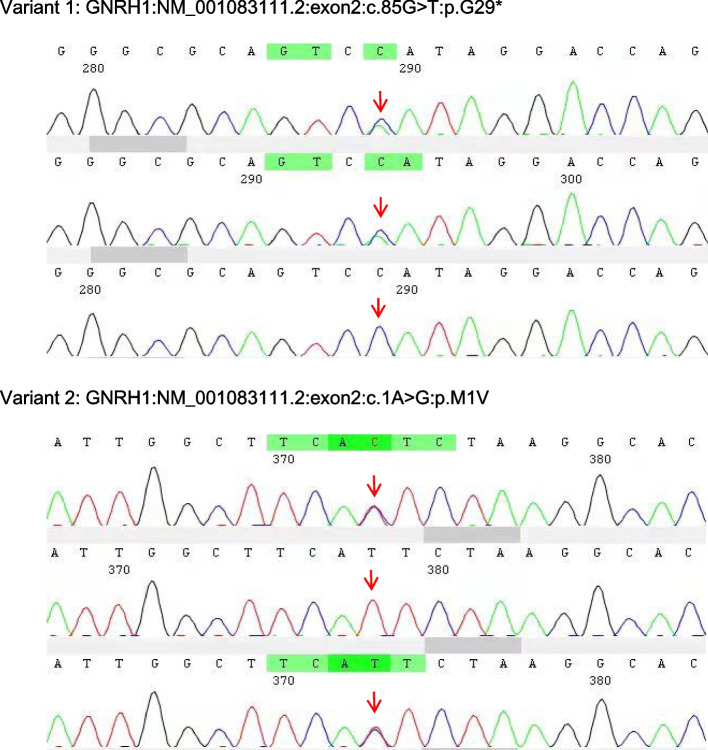
Sanger sequencing results of the patient (upper row), his father (middle row), and his mother (lower row)

**Fig. 4:**
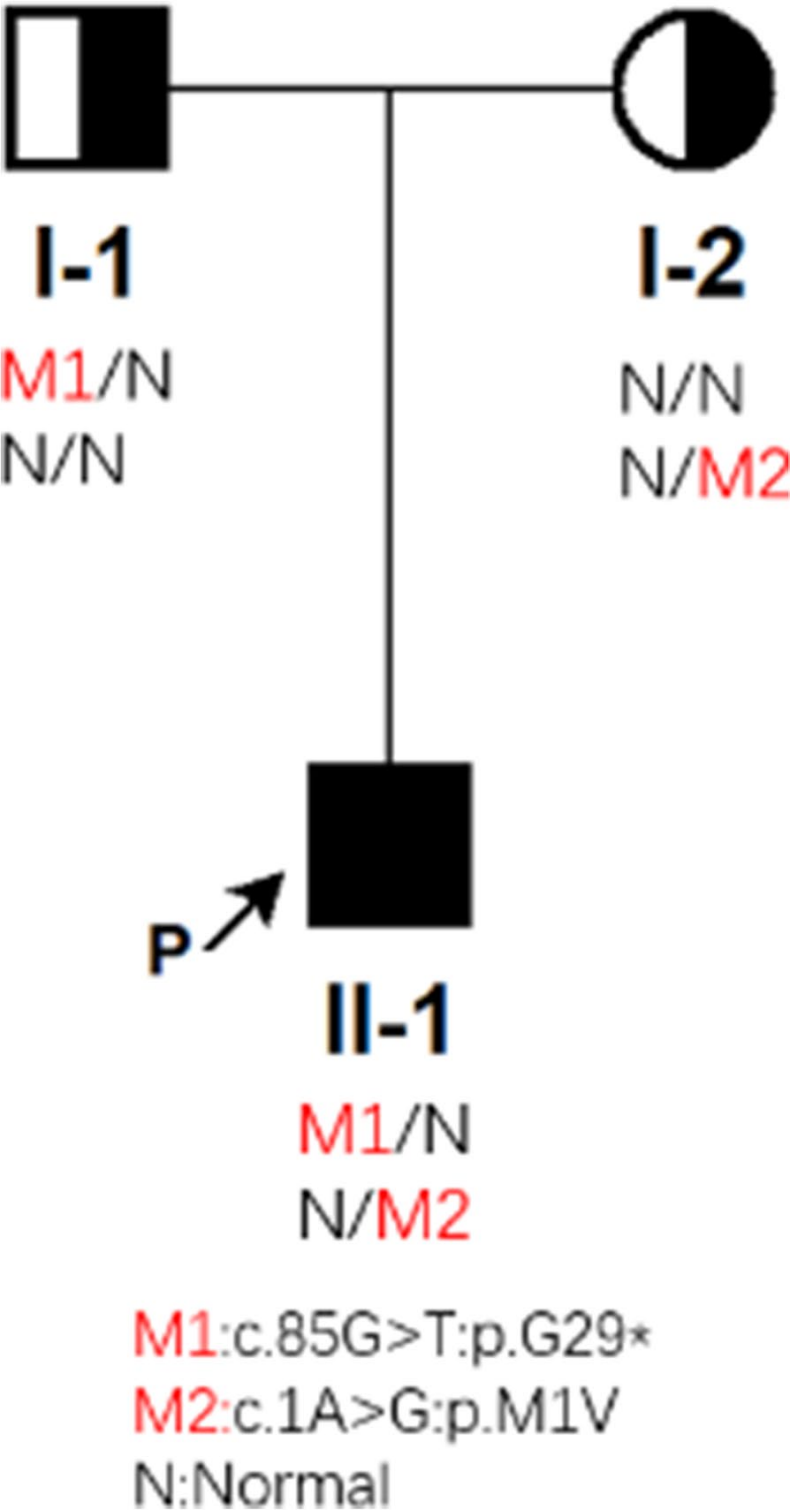
*GNRH1* gene mutations detected in the patient with idiopathic hypogonadotropic hypogonadism. I-I: father, I-2: mother, and II-1: patient

Genetic testing was performed by Westham Biomedical Technology Co. Ltd. (Shanghai, China) with the informed consent of the patient and his family. Blood samples were collected from the patient and his parents. After genomic DNA extraction, sequencing libraries were constructed. All exons and adjacent splice regions (20 bp) of the target genes were captured by the probes through hybridization and then enriched, together with the full length of the mitochondrial genome. Copy number variants were detected by high-throughput sequencing. Sequence variation data were analyzed in accordance with the American College of Medical Genetics and Genomics (ACMG) guidelines [9]. First, the raw data were quality-filtered, and any reads failing to meet the required standards were eliminated. Then, the remaining data were compared to the human reference genome hg19. The GATK software (http://www.broadinstitute.org/gatk/) was used to identify single nucleotide variants and insertion-deletion variants. The identified variants were subjected to further screening using the Genome Aggregation Database (gnomAD; http://www.gnomad-sg.org/), as well as bioinformatic analysis and prediction. Furthermore, the XHMM and CLAMMS algorithms were employed to conduct copy number variant analysis of the region covered by the probes [10, 11]. Two heterozygous variants in the *GNRH1* gene were identified, namely, nonsense variant 1: c.85G > T:p.G29* and variant 2: c.1A > G:p.M1V (Fig. 2). All genetic findings by whole-exome sequencing were confirmed by Sanger sequencing (Fig. 3). Such mutations were not found in the Genome Aggregation Database (gnomAD; http://www.gnomad-sg.org/). Variant 1 was classified as a pathogenic variant inherited from the patient's father, and variant 2 was classified as a possible pathogenic variant inherited from his mother. Together, they constituted the compound heterozygous variants in the GNRH1 gene (Fig. 4).

## Discussion

The pathogenesis of IHH is associated with hypothalamic GnRH neuronal differentiation or developmental disorders that result in inadequate GnRH secretion or secretory insufficiency [12]. The typical clinical features of male IHH patients are delayed puberty and azoospermia [13]. Delayed puberty is defined as delayed breast development in girls or delayed testicular development in boys, with a cut-off value of 13 years for girls and 14 years for boys [14]. The most common causes of delayed puberty are IHH and CDGP, which have almost identical clinical manifestations and biochemical characteristics in early puberty [15]. Distinguishing between IHH and CDGP is extremely challenging, and there is still a lack of consensus on a 'gold standard' test to reliably distinguish between the two disorders [2].

In a retrospective study of 174 boys aged 14–15 years with delayed puberty, Varimo et al. [16]. showed that testicular size could differentiate between boys with IHH and CDGP; when using a testicular volume of 1.1 mL as the threshold (clinical measurement), the test achieved 100% sensitivity and 91% specificity. At present, testicular volume is probably the most reliable parameter to distinguish IHH from CDGP in boys with delayed puberty [4]. In addition, bilateral cryptorchidism in newborns may indicate IHH [17]. Accordingly, data from a prospective study showed that in patients with IHH who exhibited bilateral cryptorchidism, spontaneous descent of the testis was unlikely to occur after 3 months of life [18]. Therefore, active screening of male infants with bilateral cryptorchidism with or without micropenis at 3 months of age may be useful for the early diagnosis of IHH. However, the mean age of diagnosis and onset of clinical treatment for IHH in male patients is often inappropriately delayed until late adolescence or early adulthood. Our patient was not diagnosed in a timely manner, and his treatment was therefore delayed.

The accepted tentative diagnostic criteria for IHH have been age ≥ 18 years, incomplete or absent puberty, serum testosterone levels < 100 ng/dl (3.5 nmol/L), normal pituitary function, and normal hypothalamic-pituitary imaging features [19]. Based on these criteria, GnRH-agonist and HCG stimulation tests can be used to identify IHH and CDGP [20]. The diagnosis of IHH can be excluded if the peak LH is > 4.3 IU/L after 60 min of intravenous administration of 100 µg gonadorelin [21]. In addition, testosterone levels > 100 ng/dl after intramuscular injection of 2000 IU HCG is indicative of good interstitial testicular function [22]. Our patient had a 10-year history of cryptorchidism and micropenis, lack of pubic or axillary hair, a 2-year delay in bone age, and reduced bone mineral density. His blood testosterone levels were low, with FSH and LH levels also below normal. According to the HCG stimulation test results, testosterone could be stimulated and there was no delay in the peak value, and this implies normal testicular interstitial cell function. Therefore, in this case, the lesion was located in the hypothalamus or pituitary gland. IHH patients are chronically deficient in GnRH, so they cannot respond to exogenous GnRH in a timely manner. In our patient, the GnRH stimulation test results showed a peak LH of < 4.3 IU/L at 60 min. Based on his clinical manifestations and biochemical test results, he was diagnosed with nIHH.

Testosterone replacement therapy is the classic treatment for hypogonadism [23]. However, the side effects of the treatment include increased red blood cell counts and premature epiphyseal closure [24]. In addition, patients need to be followed up with regular monitoring of sex hormones and observation of testicular and penile changes for a period of approximately 2 years. It is worth noting that according to a large retrospective study, up to 22% of patients with IHH may experience reversal of hypogonadotropic hypogonadism, and some of them even achieve normal sperm counts [25]. Our patient received testosterone replacement therapy and responded to the treatment. At the one-month follow-up, his penis was approximately 3.5 cm long and 7.5 cm in circumference, and two months later, it was 5 cm long and 9 cm in circumference. No side effects were observed in the ongoing follow-up.

IHH is genetically heterogeneous and can be expressed as x-linked recessive, autosomal recessive, autosomal dominant, and oligogenetic inheritance [26]. A previous genetic study has established that loss-of-function mutations in the *GNRH1* gene lead to autosomal recessive nIHH [27]. Genetic testing results indicated that our patient carried two heterozygous variants of the *GNRH1* gene. In variant 1, G was replaced with T at position 85 of the cDNA, which enabled codon 29 to encode a stop codon instead of glycine. In variant 2, A was replaced with G at position 1 of the cDNA, which enabled codon 1 to encode valine instead of methionine, thus disrupting the start codon of the *GNRH1* gene. The nonsense mutation can induce premature termination of polypeptide chain synthesis and shortening of peptide chain length, which is predicted to cause protein truncation [28]. The disruption of the start codon may lead to gene function deficiency. The compound heterozygous variants are likely to impair the function of GnRH or GnRH-related peptides, resulting in a more severe phenotype, like our nIHH patient with completely absent puberty and bilateral cryptorchidism.

To the best of our knowledge, compound heterozygous mutations in the *GNRH1* gene detected in our patient have not been reported as a cause of nIHH. This finding is therefore valuable, as the identification of novel causative mutations could enable more accurate classification of IHH-related genes and provide patients with more complete information about the diagnosis, including their family members' risk of disease development.

## Data Availability

The datasets used and/or analyzed during the current study are available from the corresponding author on reasonable request.

## Abbreviations

IHH: Idiopathic hypogonadotropic hypogonadism
nIHH: Normal olfactory function Idiopathic hypogonadotropic hypogonadism
CDGP: Constitutional delay of growth and puberty
LH: Luteinizing hormone
FSH: Follicle-stimulating hormone
GnRH: Gonadotropin-releasing hormone
HCG: Human chorionic gonadotropin

## References

[1] Topaloğlu AK (2017). Update on the genetics of idiopathic hypogonadotropic Hypogonadis-m. Clin Res Pediatr Endocrinol.

[2] Bianco SD, Kaiser UB (2009). The genetic and molecular basis of idiopathic hypogonadotro-pic hypogonadism. Nat Rev Endocrinol.

[3] Mitchell AL, Dwyer A, Pitteloud N, Quinton R (2011). Genetic basis and variable phenotypic expression of Kallmann syndrome: towards a unifying theory. Trends Endocrinol Metab.

[4] Young J, Xu C, Papadakis GE (2019). Clinical Management of Congenital Hypogona-dotropic Hypogonadism. Endocr Rev.

[5] Chan YM, de Guillebon A, Lang-Muritano M (2009). GNRH1 mutations in patients w-ith idiopathic hypogonadotropic hypogonadism. Proc Natl Acad Sci U S A.

[6] Chan YM (2011). A needle in a haystack: mutations in GNRH1 as a rare cause of isolat-ed GnRH deficiency. Mol Cell Endocrinol.

[7] Marshall WA, Tanner JM (1970). Variations in the pattern of pubertal changes in boys. Arch Dis Child.

[8] Viswanathan V, Eugster EA (2011). Etiology and treatment of hypogonadism in adolescents. Pediatr Clin North Am.

[9] Richards S, Aziz N, Bale S (2015). Standards and guidelines for the interpretation of sequence variants: a joint consensus recommendation of the American College of Medical Genetics and Genomics and the Association for Molecular Pathology. Genet Med.

[10] Fromer M, Moran JL, Chambert K (2012). Discovery and statistical genotyping of copy-number variation from whole-exome sequencing depth. Am J Hum Genet.

[11] Packer JS, Maxwell EK, O'Dushlaine C (2016). CLAMMS: a scalable algorithm for calling common and rare copy number variants from exome sequencing data. Bioinformatics.

[12] Fraietta R, Zylberstejn DS, Esteves SC (2013). Hypogonadotropic hypogonadism revisited. Clinics.

[13] Mao JF, Xu HL, Duan J (2015). Reversal of idiopathic hypogonadotropic hypogonad-ism: a cohort study in Chinese patients. Asian J Androl.

[14] Zhu J, Chan YM (2015). Fertility issues for patients with hypogonadotropic causes of delayed puberty. Endocrinol Metab Clin North Am.

[15] Bonomi M, Vezzoli V, Krausz C (2018). Characteristics of a nationwide cohort of patients presenting with isolated hypogonadotropic hypogonadism (IHH). Eur Endocrinol.

[16] Varimo T, Miettinen PJ, Kansakoski J (2017). Congenital hypogonadotropic hypogonadism, functional hypogonadotropism or constitutional delay of growth and puberty? An analysis of a large patient series from a single tertiary center. Hum Reprod.

[17] Pitteloud N, Hayes FJ, Boepple PA (2002). The role of prior pubertal development, biochemical markers of testicular maturation, and genetics in elucidating the phenotypic heterogeneity of idiopathic hypogonadotropic hypogonadism. Clin Endocrinol Metab.

[18] Sijstermans K, Hack WW, Meijer RW (2008). The frequency of undescended testis from birth to adulthood: a review. Int J Androl.

[19] Raivio T, Falardeau J, Dwyer A (2007). Reversal of idiopathic hypogonadotropic hyp-ogonadism. N Engl J Med.

[20] Segal TY, Mehta A, Anazodo A (2009). Role of gonadotropin-releasing hormone and human chorionic gonadotropin stimulation tests in differentiating patients with hypogonadotropic hypogonadism from those with constitutional delay of growth and puberty. Clin Endocrinol Metab.

[21] Grinspon RP, Ropelato MG, Gottlieb S (2010). Basal follicle-stimulating hormone and peak gonadotropin levels after gonadotropin-releasing hormone infusion show high diagnostic accuracy in boys with suspicion of hypogonadotropic hypogonadism. Clin Endocrinol Metab.

[22] Dunkel L, Perheentupa J, Sorva R (1985). Single versus repeated dose human chorionic gonadotropin stimulation in the differential diagnosis of hypogonadotropic hypogonadism. Clin Endocrinol Metab.

[23] Boehm U, Bouloux PM, Dattani MT (2015). Expert consensus document: European Consensus Statement on congenital hypogonadotropic hypogonadism–pathogenesis, diagnosis and treatment. Nat Rev Endocrinol.

[24] Finkelstein JS, Klibanski A, Neer RM (1989). Increases in bone density during treatment of men with idiopathic hypogonadotropic hypogonadism. Clin Endocrinol Metab.

[25] Sidhoum VF, Chan YM, Lippincott MF (2014). Reversal and relapse of hypogonadotropic hypogonadism: resilience and fragility of the reproductive neuroendocrine system. Clin Endocrinol Metab.

[26] Bhagavath B, Ozata M, Ozdemir IC (2005). The prevalence of gonadotropin-releasing hormone receptor mutations in a large cohort of patients with hypogonadotropic hypogonadism. Fertil Steril.

[27] Maione L, Albarel F, Bouchard P (2013). R31C GNRH1 mutation and congenital hypogonadotropic hypogonadism. PLoS ONE.

[28] Mason AJ, Hayflick JS, Zoeller RT (1986). A deletion truncating the gonadotropin-releasing hormone gene is responsible for hypogonadism in the hpg mouse. Science.

